# Proterozoic supercontinent break-up as a driver for oxygenation events and subsequent carbon isotope excursions

**DOI:** 10.1093/pnasnexus/pgac036

**Published:** 2022-03-30

**Authors:** James Eguchi, Charles W Diamond, Timothy W Lyons

**Affiliations:** Department of Earth and Planetary Sciences, University of California, Riverside, CA 92521, USA; Department of Earth and Planetary Sciences, University of California, Riverside, CA 92521, USA; Department of Earth and Planetary Sciences, University of California, Riverside, CA 92521, USA

**Keywords:** oxygen, supercontinent, carbon, isotopes, subduction

## Abstract

Oxygen and carbon are 2 elements critical for life on Earth. Earth's most dramatic oxygenation events and carbon isotope excursions (CIE) occurred during the Proterozoic, including the Paleoproterozoic Great Oxidation Event and the associated Lomagundi CIE, the Neoproterozoic Oxygenation event, and the Shuram negative CIE during the late Neoproterozoic. A specific pattern of a long-lived positive CIE followed by a negative CIE is observed in association with oxygenation events during the Paleo- and Neo-proterozoic. We present results from a carbon cycle model designed to couple the surface and interior cycling of carbon that reproduce this pattern. The model assumes organic carbon resides in the mantle longer than carbonate, leading to systematic temporal variations in the δ^13^C of volcanic CO_2_ emissions. When the model is perturbed by periods of enhanced continental weathering, increased amounts of carbonate and organic carbon are buried. Increased deposition of organic carbon allows O_2_ accumulation, while positive CIEs are driven by rapid release of subducted carbonate-derived CO_2_ at arcs. The subsequent negative CIEs are driven by the delayed release of organic C-derived CO_2_ at ocean islands. Our model reproduces the sequences observed in the Paleo- and Neo-proterozoic, that is oxygenation accompanied by a positive CIE followed by a negative CIE. Periods of enhanced weathering correspond temporally to supercontinent break-up, suggesting an important connection between global tectonics and the evolution of oxygen and carbon on Earth.

Significance Statement The Proterozoic hosts Earth's most dramatic oxygenation events and CIEs. Using a geochemical model that considers the links between the Earth's surface and interior carbon cycles, we demonstrate that systematic changes in δ^13^C of volcanic outgassing can reproduce the major CIEs of marine carbonates for nearly the entirety of Earth history, while also capturing the broad trends of atmospheric oxygen evolution. Systematic changes in δ^13^C of volcanic outgassing are the result of longer mantle residence times of graphitized organic carbon compared to carbonate. Timings of model perturbations correspond to supercontinent break-ups, which suggests that supercontinent break-ups are major drivers of atmospheric oxygenation and subsequent positive and negative CIEs.

## Introduction

**Fig. 1.**
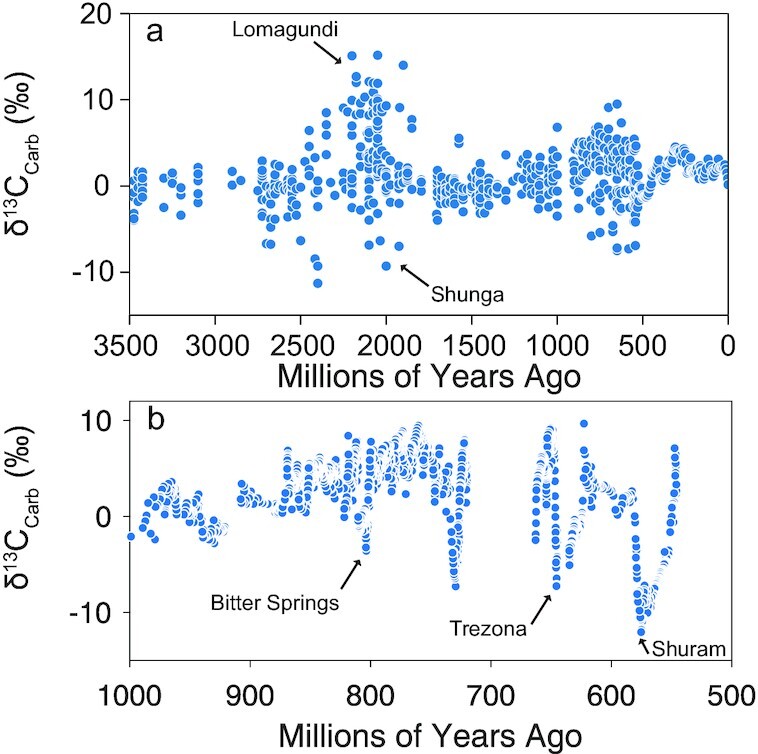
Carbon isotope record for marine carbonates with major CIEs described in the text. (a) Compilation of δ^13^C_carb_ for Archean to present (from 8). (b) Compilation of δ^13^C_carb_ for Neoproterozoic (from 74) showing the broad positive CIE punctuated by negative CIEs.

Carbon and oxygen play critical roles in the story life on Earth. The cycling of these 2 elements is intimately linked through oxygenic photosynthesis and aerobic respiration. These links are evident in the geologic record, particularly during the Proterozoic. The Paleoproterozoic hosts the Great Oxidation Event (1–3) and the associated Lomagundi carbon isotope excursion (CIE) (4) (Figure 1a), while the Neoproterozoic hosts the Neoproterozoic Oxygenation Event (5–7) with an associated broad positive CIE (8) (Figure 1a and b). The association between changing atmospheric oxygen and the carbon isotope record is a result of oxygenic photosynthesis and the burial of organic matter (9, 10). Free oxygen accumulates in the atmosphere when organic carbon is produced during oxygenic photosynthesis and subsequently buried, which precludes oxygen consumption through aerobic respiration. Organic C produced during oxygenic photosynthesis is depleted in ^13^C, leaving the global reservoir from which marine carbonates precipitate enriched in ^13^C. Therefore, when the ratio of carbon buried as organic C relative to inorganic carbonate (*f*_org_) increases, atmospheric oxygen levels increase, and δ^13^C values of marine carbonates (δ^13^C_carb_) increase concomitantly. This biological link between oxygen and carbon, 2 elements essential to Earth's habitability, has led to speculation on the mechanisms controlling their evolutionary history (9–11).

In addition to extreme positive CIEs, the Proterozoic also hosts some of the largest negative CIEs in the geologic record (12). According to the conventional view of the carbon cycle, decreased *f*_org_ can cause negative CIEs. However, the extreme magnitude of some Proterozoic negative CIEs, such as the Ediacaran Shuram anomaly, reach δ^13^C_carb_ of ∼ −15‰ (13) (Figure 1b), making them difficult to explain using the conventional mechanisms of changing *f*_org_. To explain these extremes for Proterozoic negative CIEs, authors have invoked perturbations to the global carbon cycle that are not seen on the modern Earth, such as the large-scale oxidation of reduced forms of carbon—dissolved organic matter and methane in particular (14–16). Another suggestion is that Proterozoic negative CIEs could be the result of authigenic carbonate production, acting either at the local or global scale (17, 18). Other researchers have questioned the primary nature of large negative CIEs and propose that they are the result of local alteration during burial (19), or that they record local shallow water processes rather than large changes to the global carbon cycle (20).

Due to their temporal association with critical points of biological evolution (13), it is important to understand if extreme negative CIEs, such as the Shuram, are primary signals in the δ^13^C record. An argument against a primary origin for these large negative CIEs is that they are inconsistent with the way the modern C cycle behaves and, therefore, require extreme perturbations to the ocean–atmosphere C cycle (14–16). If the main input of CO_2_ into the ocean–atmosphere system, volcanic CO_2_ outgassing, is constant at ∼ −5 ‰, it becomes difficult for modern global carbon cycle processes to shift δ^13^C_carb_ to values as low as ∼ −15 ‰ (21, 22). However, the assumption that the δ^13^C of global volcanic CO_2_ (δ^13^C_volc_) emissions has remained constant at ∼ −5 ‰ throughout Earth history may be incorrect (11, 23, 24). Large quantities of surficial C are subducted into the Earth's interior, and much of that subducted C is recycled back to the surface through volcanism (25–27). We know that δ^13^C of surficial C reservoirs have changed through time (Figure 1), therefore, it may be reasonable to assume that δ^13^C_volc_, which is likely to be heavily influenced by subducted surficial C (26, 28), has also changed through time. We also note that differential weathering of continentally derived carbonates and organic C has the potential to change the δ^13^C of C entering the ocean. However, oxygen levels may act to regulate the relative weathering fluxes of the 2 continental C reservoirs, thus acting as a feedback that effectively prevents weathering of continentally derived C from driving long-lived CIEs in marine carbonates (29). If correct, we are left with volcanic outgassing as the primary flux capable of altering the isotopic composition of C entering the ocean–atmosphere system. Here, we employ a novel carbon cycle model that accounts for variations in δ^13^C_volc_ to test whether they can explain the positive and negative CIEs of the Proterozoic, thereby providing a new mechanism for the extreme CIEs of the Proterozoic as primary signals of perturbations to the global C cycle.

We begin by providing a brief, qualitative overview of how the model predicts coupled oxygenation events and CIEs (see Materials and Methods for detailed model description). The burial flux of C is controlled by the weathering of silicates, which depends on atmospheric CO_2_ levels and the weatherability of silicate minerals exposed on the continent:

(1) $$\begin{equation*}
{{\rm{F}}_w} = k \times \,[ {{\rm{C}}{{\rm{O}}_{{\rm{2,atm}}}}}]{\rm{,}}
\end{equation*}
$$

where F_w_ is the weathering flux of silicate rocks, *k* is a scalar, which represents the strength of the weathering feedback (30), and [CO_2, atm_] is the concentration of CO_2_ in the atmosphere. Therefore, any event that increases *k* or [CO_2, atm_] has the potential to increase the weathering flux of continental silicate rocks. An enhanced silicate weathering flux will increase the flux of cations such as Ca^2+^ to the ocean, increasing the precipitation and burial of carbonates. Continental weathering also delivers key nutrients such as P to the oceans (31). Therefore, enhanced carbonate burial is likely to be accompanied by enhanced organic C burial and increased atmospheric O_2_. If the burial flux of carbonate and organic C increase in equal proportion, then atmospheric oxygen levels can increase with no change in *f*_org_ and consequently no change in δ^13^C_carb_. Therefore, atmospheric oxygen may increase with no immediate change to δ^13^C, offering a possible explanation for the apparent delay between initial accumulation of O_2_ at the onset of the Great Oxidation Event and the rising limb of the Lomagundi CIE (3, 4).

Following enhanced burial of C on the seafloor, some fraction of carbonate and organic matter will be subducted into the mantle, while the remainder will be deposited on continental shelves and become part of the continental inventory through continental collision and uplift. How we treat carbonate and organic C in the mantle, and specifically their assumed distinct behaviors, is a novel aspect of our model. Central to our results, the model assumes that organic C has a longer residence time in the mantle than carbonate. Under subarc conditions, inorganic carbonate is more easily transported out of the slab, as compared to graphitized organic C, due to its higher solubility in fluids and melts (24, 26, 32, 33). This behavior has been observed in subducted lithologies, which show evidence for significant carbonate loss, while retaining significant amounts of graphitized organic C (34). Therefore, the CO_2_ emitted at arc volcanoes is likely to be preferentially sourced from subducted carbonates compared to organic C. The possibility of preferential carbonate release at arc volcanoes may be supported by the observation that on a global scale, the flux-weighted mean of δ^13^C_volc_ at arc volcanoes is ≈ −3 ‰ (23), which is heavier than δ^13^C_volc_ at mid-ocean ridges, which are thought to be dominated by primordial mantle C.

Subducted carbonates may be recycled back to the surface via arc volcanism on timescales of tens of millions of years (35). Therefore, some tens of millions of years after the increased burial of carbonate and organic matter at the surface, there will be increased outgassing of carbonate-derived CO_2_ at arc volcanoes, thus tipping the balance of surface inputs toward returning carbonate C to the surface and away from returning organic C. In our model, this step initiates the rising limb of a positive CIE. Release of subducted carbonate C, although a fundamentally different process, is analogous to carbonate platform weathering, which similarly returns carbonate C to the system and has been invoked as a mechanism for driving the Late Ordovician positive CIE (36).

After carbonate C is released at subarc depths, the subducting slab will be enriched in organic C relative to inorganic (carbonate) C as it descends deeper into the mantle. Low δ^13^C values recorded in eclogitic diamonds may provide evidence for the deep subduction and retention of low δ^13^C values in subducted organic C (37). The slab may then become entrained in upwelling mantle plumes and melt as it nears the surface, releasing organic C-derived CO_2_ at plume-fed volcanoes, such as ocean island centers. Although the isotopic composition of CO_2_ emitted at ocean islands remains poorly constrained, there are suggestions that CO_2_ in samples from the Pitcairn hotspot may be isotopically light compared to mid-ocean ridges (38). Additionally, a recent study on kimberlites, which may be derived from mantle plumes similar to ocean island basalts, showed that δ^13^C in many Phanerozoic examples exhibit low values, which the authors attribute to subducted organic C (39). The model assumes that organic C is released on the order of hundreds of millions of years after subduction, corresponding to the timescale of convective overturn in the mantle (24, 40). Radiogenic isotopes have also been used to suggest that crustal recycling at Mauna Loa occurs on the order of 200–650 million years (41). It follows that the enhanced flux of subducted organic C will be released at ocean island volcanoes on the order of hundreds of millions of years after the initial C burial event at the surface, and the release of organic C-derived CO_2_ will decrease δ^13^C_volc_, abruptly terminating the long-lived positive CIE and driving the negative CIE.

In sum, in response to a strengthened silicate weathering feedback, the model predicts atmospheric oxygenation driven by enhanced burial of organic matter contemporaneously with increased carbonate burial. This trigger and the corresponding impacts on the carbon cycle are associated with the onset of a long-lived positive CIE due to the preferential release of carbonate C at arcs. Isotopically heavy carbonates will continue to be deposited and subducted until the initial pulse of subducted organic matter is returned to the surficial system at ocean islands, terminating the positive CIE and driving a negative CIE. In the following section, we investigate whether the model can reproduce the series of oxygenation events and CIEs of the Proterozoic.

## Results

**Fig. 2.**
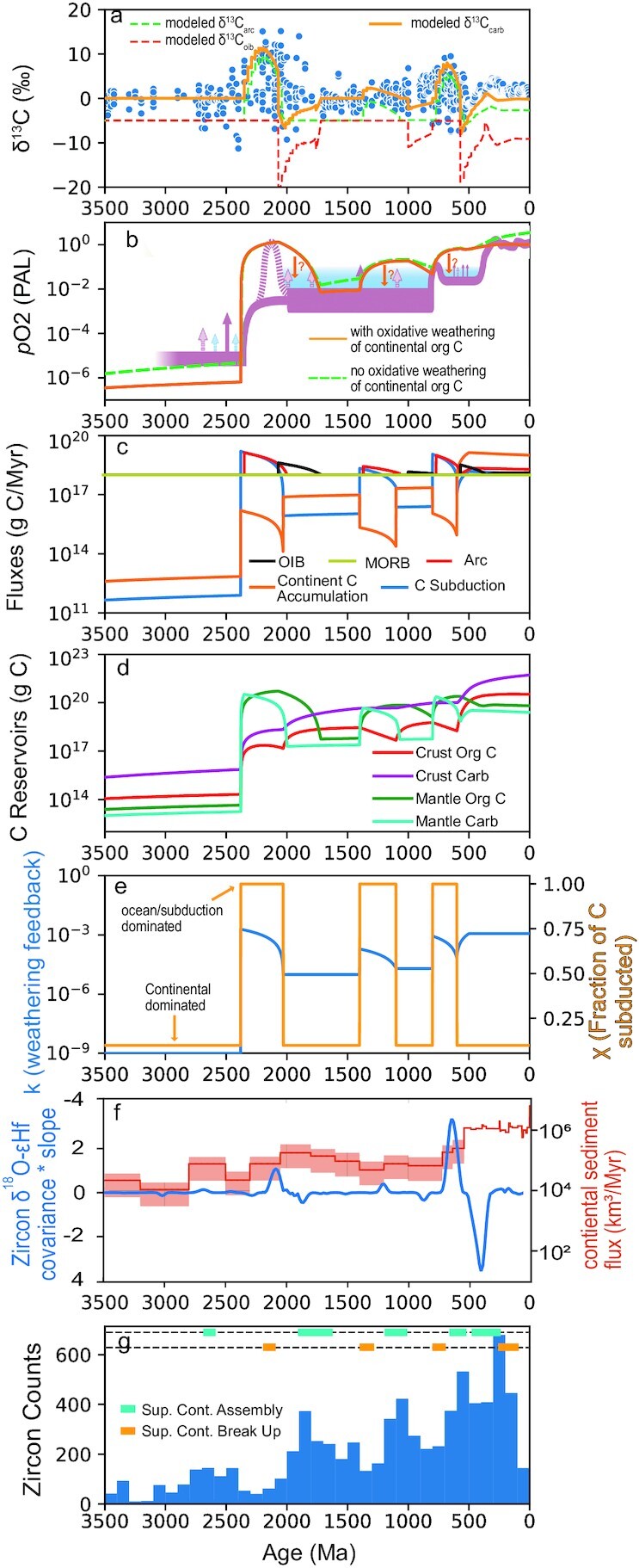
Model results for atmospheric oxygen and δ^13^C of marine carbonates compared with natural data. (a) δ^13^C of marine carbonates and volcanic centers versus time. Blue data points are natural data from Krissansen-Totton et al. (8). Orange curve is model result for carbonates, red and green curves are modeled δ^13^C for arcs and ocean islands, respectively. (b) Atmospheric oxygen level (PAL) through time. Pink curve and blue-shaded region is proposed evolution of atmospheric oxygen based on various proxies (7). See (7) for detailed explanation of proxy curve. Orange curve is model result with oxidative weathering of crustal organic C and green-dashed curve is model result without oxidative weathering of crustal organic C. Orange downward pointing arrows with question marks were included to illustrate possible lower levels of O_2_ if a complete consideration of oxygen sinks was included. (c) Modeled C fluxes from different volcanic settings and the C drawdown flux from atmosphere driven by silicate weathering. (d) Modeled C reservoirs through time. (e) Prescribed values of *k* (strength of silicate weathering feedback through time) and χ (fraction of buried C that is subducted) through time. *k* and χ are the drivers of all perturbations in the model. (f) Blue curve is covariance times the slope of δ^18^O-εHf in zircons from Keller et al. (53). Peaks in this curve are suggested to signal periods of enhanced subduction of continental weathering products. Orange curve is estimated continental sediment flux through time from Husson and Peters (56). (g) Frequency of zircons through time from Tang et al. (60) and times of supercontinent break-up and assembly from Condie and Aster (49).

The Proterozoic begins with the Great Oxidation event and associated Lomagundi positive CIE (Figure 1a and b) (3), which are directly followed by the Shunga negative CIE (42–44) (Figure 1b). This sequence of events is identical to the generic model scenario described above. To generate the model run shown in Figure 2, the only parameters that change are *k*, which accounts for the strength of the silicate weathering feedback, and χ, which controls the fraction of buried carbon that is subducted into the mantle. We note that *f*_org_ remains at 0.20 for each model run, so all CIEs in the simulations result solely from changes in δ^13^C_volc_. At 2.4 Ga, we prescribe an instantaneous increase in *k*, followed by a linear decay over a time span dictated by tectonic cycles (described below; Figure 2e). The increase in *k* is coupled with an immediate transition to a higher value of χ that persists for the duration of increased *k* (Figure 2e). This combination simulates increased continental weathering accompanied by a rapid shift in the location of C deposition from dominantly on continental shelves to dominantly on oceanic crust. The rationale behind our decisions to increase *k* and χ will be discussed below. This perturbation causes an initial spike in the weathering flux, increasing the size of the crustal and mantle reservoirs of organic C, which increases atmospheric O_2_ (Figure 2b–d). Oxygen increases initially, but decays when organic C is released as CO_2_ at ocean islands (Figure 2b and c). Release of organic C-derived CO_2_ at ocean islands decreases atmospheric O_2_ in the model because we assume that every mole of organic C that is oxidized and degassed as CO_2_ leads to the consumption of 1 mole of O_2._ The reduction of chemical species (likely Fe) results from the oxidation of organic C to CO_2_. This reduced chemical species will be erupted in association with ocean island basalts, along with the CO_2_, and will consume atmospheric O_2_ when oxidized. This process is analogous to aerobic respiration in terms of the net consequences and has been previously recognized in C cycle redox models (45). The modeled perturbation of increased *k* and χ predicts increased organic C and carbonated deposition across the Archean–Proterozoic boundary (Figure 2d). Consistent with this suggestion, a transition from very little marine carbonate precipitation in the Archean to appreciable amounts in the Proterozoic (Figure 2d) has been identified in a recent compilation of carbonate formations through time (46). Increased depositional fluxes of organic C across the Archean–Proterozoic boundary has also been recognized in the continental sedimentary record (47).

A few tens of millions of years after the initial increase in *k* and χ, the arc CO_2_ flux and δ^13^C_arc_ increase, causing δ^13^C_volc_ and in turn δ^13^C_carb_ to rise (Figure 2a and c). Over the next few hundred million years, the arc CO_2_ flux decays, so that by the time the increased flux of organic C is released at ocean islands, the ocean island CO_2_ flux with low δ^13^C (Figure 2a) dominates global volcanic CO_2_ inputs (Figure 2c). This progression leads to a rapid decrease in δ^13^C_volc_ and a relatively abrupt transition from a positive CIE to a negative CIE (Figure 2a).

Following the CIEs in the Paleoproterozoic, δ^13^C_carb_ is relatively stable until around 1.4 Ga (8) (Figure 2a), marked by the initiation of a broad positive CIE that persists until the Ediacaran (Figure 2a). In the model, the duration of positive CIEs is controlled by the residence time of organic C in the mantle (24). The broad positive CIE at the end of the Proterozoic is significantly longer than the Lomagundi CIE, lasting close to ∼ 1 Gyrs (Figure 2a). Therefore, unless mantle convection slowed significantly, it may be difficult to explain the events at the end of the Proterozoic via the same mechanism invoked for the Paleoproterozoic. However, this broad positive CIE in the late Proterozoic is not consistently positive but instead is punctuated by excursions to near-zero and sometimes negative δ^13^C values (Figure 2a). Here, we test whether prescribing 2 discrete events during the second half of the Proterozoic can reproduce the observed late Proterozoic pattern. Below, we discuss how supercontinent cycles may trigger the enhanced continental weathering events required in the model to reproduce the observed series of oxygenation events and CIEs, and why the event in the Neoproterozoic may be a composite of 2 distinct events rather than 1.

To test the idea that the broad positive CIE at the end of the Proterozoic is a composite event, we prescribe the first of 2 increases in *k* and χ at 1.4 Ga (Figure 2e). The prescribed increase in *k* is relatively small, so the model predicts correspondingly small CIEs and a small increase in atmospheric oxygen (Figure   2b), which may be consistent with a proposed transient oxygenation event in the Mesoproterozoic (48). We prescribe a second and larger increase in *k* and χ at ∼800 Ma (Figure 2e). The increased *k* and χ cause a spike in the burial flux of both carbonate and organic C (via weathering; Figure 2c), leading to a second major step in atmospheric O_2_ levels (Figure 2b). The possibility of significantly increased atmospheric O_2_ at 800 Ma is reviewed in Lyons et al. (7). This rise in O_2_ is accompanied by a continuation of the broad positive CIE that lasts until around 550 Ma, when it is terminated by an extremely negative CIE (Figure 2a) corresponding in the model to a spike in the ocean island CO_2_ flux (Figure 2c). The negative CIE, represented in the geologic record by the Ediacaran Shuram anomaly, is relatively short-lived and recovers rapidly and stabilizes at near-zero values (Figure 2a). Thus, we see a repeat of the sequence of an oxygenation event closely accompanied by a positive CIE terminated by a negative CIE. The prescribed increases in *k* and χ at 800 Ma brings modeled oxygen levels to a peak of ∼ 0.5 present atmospheric level (PAL) by ∼550 Ma (Figure 2b). We emphasize that the purpose of the model is not to closely reproduce atmospheric O_2_ levels (especially in the Phanerozoic), but rather to broadly reproduce the increases in atmospheric O_2_ that coincide with positive CIEs followed by negative CIEs. However, to illustrate that our model is not drastically inconsistent with present day O_2_ levels of 1 PAL, we increase *k* into the Phanerozoic, so that modeled O_2_ levels reach ∼1 PAL (Figure 2b and e). Due to low χ after 550 Ma (Figure 2e), the modeled increase in Phanerozoic C burial occurs mostly on the continents and, therefore, exerts little effect on modeled δ^13^C (Figure 2a).

We acknowledge that the prescribed changes in *k* are large (Figure 2e); however, they may coincide with extreme events that may have drastically affected silicate weathering. Caves et al. (30) found that *k* may have varied by as much as a factor of 3 during the Cenozoic, which was likely tectonically quiescent compared to the supercontinent break-ups (49) and proposed rapid emergence of continents from the oceans (50–52) that helped define the Paleoproterozoic and Neoproterozoic. Additionally, proposed proxies for recycling of continental weathering products show extreme signals during the Paleoproterozoic and Neoproterozoic when compared to the Cenozoic (53) (Figure 2f). Therefore, we argue that the changes in *k* and χ prescribed here may not be unreasonable and are supported by the geologic record (Figure 2f). We emphasize that the sole driver of all oxygenation events and CIEs in the present model were changes to *k* and χ, and the resulting systematic variations in δ^13^C_volc_; *f*_org_ was held constant at 0.20 throughout the model run. This approach is an obvious oversimplification, but it highlights the fact that the long-term, first-order trends of the δ^13^C_carb_ record can be reproduced by systematic changes in δ^13^C_volc_ without any changes to the fraction of carbon buried as organic matter.

## Discussion

It is striking that with only simple assumptions about differential residence times of carbonate and organic C in the mantle (24) and prescription of 3 discrete weathering events (Figure 2e), the model reproduces the broad trends of δ^13^C_carb_ and major CIEs (Figure 2a) for nearly the entirety of Earth history. Additionally, the model does a reasonable job of capturing the major trends in oxygenation and associated O_2_ events suggested by proxy data (7) (Figure 2b). However, we note that this is not intended to be an accurate model for atmospheric O_2_ and may overestimate O_2_ levels during the Proterozoic because it does not account for pyrite oxidation, nor does it account for the oxidation of reduced metamorphic and volcanic gases, all of which have been demonstrated to be important in regulating atmospheric oxygen levels (54, 55). To illustrate the potential effects oxygen sinks may have on the model, we plot model runs with and without a simple formulation for the oxidative weathering of organic C (55) (Figure 2b). Figure 2(b) shows that the inclusion of organic C weathering predicts lower O_2_ levels. Therefore, a more complete consideration of oxygen sinks should be considered but is beyond the scope of the current work.

Despite some differences in magnitudes, the general agreement of our modeled δ^13^C_carb_ and O_2_ with the geologic record motivates us to ask whether there is justification for these 3 prescribed changes to *k* and χ in the model, and indeed there is. Figure 2(e) and (f) shows that the 3 discrete weathering events prescribed in the model coincide with increases in the covariance of zircon δ^18^O-εHf, which have been attributed to enhanced subduction of continental weathering products (53). The peaks in the zircon δ^18^O-εHf are followed by peaks in the continental sediment flux, with the delay between the 2 records being more evident for the Neoproterozoic when a better continental record is preserved (53, 56) (Figure 2f). The delay between zircon δ^18^O-εHf and the continental sediment flux record may reflect a shift in the dominant location of marine sedimentation from oceanic crust, where it can be subducted to produce a peak in the δ^18^O-εHf data, to continental shelves where it can be preserved (53, 56). This shift is reflected in our model by increasing χ along with *k* at the initiation of an enhanced weathering event and keeping it elevated until the enhanced weathering ends (Figure 2e). This sequence of events is potentially consistent with what would be expected during the breakup of a supercontinent as discussed below.

Supercontinent cycling has previously been proposed as a potential control on continental weathering and atmospheric oxygen (10, 57–59). For example, Campbell and Allen (57) proposed supercontinent formation and accompanying mountain uplift as the main driver of major CO_2_ drawdown and oxygenation events. Here, we invoke supercontinent break-up as the major driver because all 3 of our proposed weathering events appear to initiate in phase with troughs in the zircon frequency record (60), which have been ascribed to supercontinent break-up (Figure 2g) (49).

**Fig. 3.**
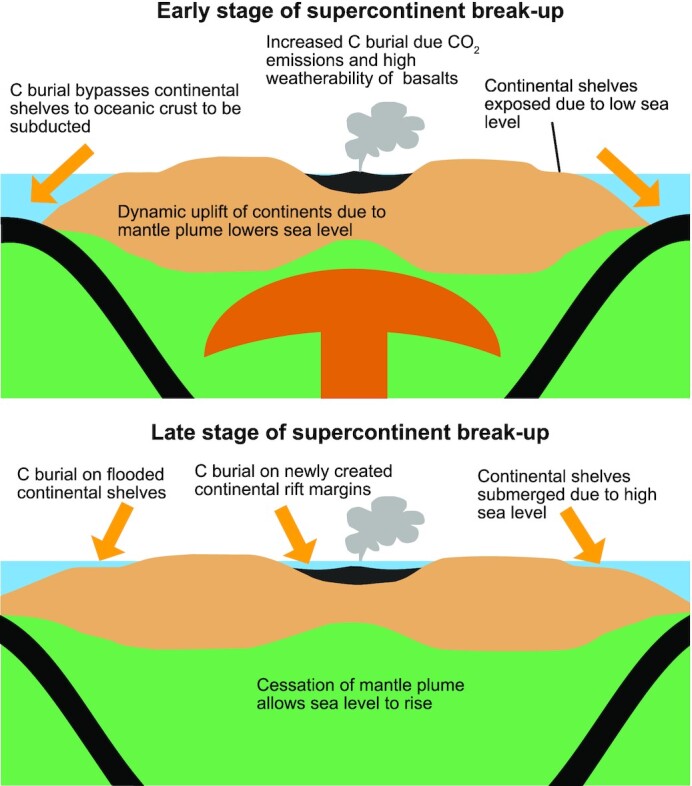
Schematic diagram showing how supercontinent break-up can increase continental weathering and C deposition. Early stage of supercontinent break-up is associated with dynamic uplift and lowered sea level due to the presence of a mantle plume. Eruption of basalts increase continental weathering due to CO_2_ emissions and weatherability of basalts. Lowered sea level allows the location of C burial to be dominated by oceanic crust, which will be subducted. Late stage of supercontinent break-up will generate rifted margins, which are primary environments for C burial. Late stage of supercontinent break-up will also have higher sea level due to cessation of the mantle plume. This will flood continental shelves, allowing the location of C burial to be dominated by continental shelves.

There are several reasons that supercontinent break-up can lead to enhanced carbonate and organic C deposition and subduction. First, the initial stages of supercontinent break-up are often associated with the eruption of large volumes of basaltic lavas (61–64), which are highly weatherable (65) and can increase global fluxes of cations and nutrients to the ocean, stimulating carbonate precipitation and organic C burial. Eruptions of basalts will also be accompanied by the outgassing of large amounts CO_2_, which can also increase continental weathering fluxes, stimulating carbon burial and subsequent CO_2_ drawdown (66) (Figure 3). Sea level is likely to be low during initial stages of supercontinent break-up due to dynamic uplift of supercontinents driven by arrival of mantle plumes (67, 68), which will allow sediments to bypass continental shelves and be deposited on oceanic crust where they can be subducted (Figure 3). The intense CO_2_ drawdown caused by initial stages of supercontinent rifting have also been proposed to be possible drivers of global glaciation (69), which can further lower sea level, exposing continental shelves and allowing for the large amounts of sediments derived from continental denudation to be deposited on oceanic crust where they can be subducted (53). In fact, the zircon δ^18^O-εHf data have been suggested to reflect extreme sediment subduction fluxes as a result of global glaciation (53). Therefore, we envision the initial stages of supercontinent break-up to increase C burial fluxes and for most of that burial to occur on oceanic crust where sediment will be subducted (53) (Figure 3), as simulated by our model perturbations of increased *k* accompanied by increased in χ (Figure 2e).

As supercontinent break-up reaches later stages, greater continental fragmentation increases the length of continental margins, thus increasing the global area of nearshore environments (70), which are the primary loci for both carbonate and organic C deposition (71) (Figure 3). Later stages of supercontinent break-up will generate new rifted basins, which are efficient traps for sediment and can favor large-scale C burial along continental margins. Additionally, smaller and more dispersed continents, in contrast to a supercontinent, have continental interiors that are closer to oceanic sources for precipitation, increasing continental area exposed to rainfall, thus increasing global continental weathering fluxes, leading to enhanced carbon burial (72). As the mantle plumes that drive the initial stages of supercontinent break-up subside and glaciers melt, sea level will rise (67), flooding continental margins, allowing sedimentation to shift from oceanic crust to continental shelves (Figure 3). Therefore, in the later stages of supercontinent break-up following the cessation of global glaciation, enhanced C burial is more likely to be accommodated in environments that become part of the continental reservoir (56, 73). Figure 2(f) shows that the continental sediment accumulation rate transitions from decreasing with time to increasing with time during supercontinent break-ups (56). However, as noted above, the peak in the subduction flux (zircon δ^18^O-εHf data) (53) occurs prior to the peak in the continental sediment flux (47) (Figure 2a), suggesting that initial stages of supercontinent break-up, and perhaps accompanying global glaciation, favor sedimentation on oceanic crust, while later stages of supercontinent break-up favor C burial on continental shelves (53, 56, 73) (Figure 3). In the model, this sequence is simulated by a transition to lower χ at the later stage of increased *k* (Figure 2e). Therefore, we propose that supercontinent break-up drives enhanced weathering and C burial, with early stages of break-up favoring C subduction, while later stages favor C accumulation on continents. The initial stages of enhanced subduction of C drive CIEs through changes in δ^13^C_volc_ driven by the differential mantle cycling of carbonates and organic C, while later stages of C accumulation on continents serve to stabilize O_2_ levels (56).

The Proterozoic witnessed dramatic transitions in surface chemistry, biology, and tectonics. Our results suggest that major supercontinent break-up may have played a key role multiple times in the biogeochemical evolution of Earth with predictable and repeatable consequences that can be recorded in the geologic record as enhanced continental weathering resulting from supercontinent break-up, increased atmospheric oxygen through organic C burial, and a subsequent positive CIE followed by a negative CIE driven by variable residence times of carbonate and organic C in the mantle. We acknowledge that the primary nature of extreme negative CIEs, such as the Shunga in the Paleoproterozoic and the Shuram in the Neoproterozoic, is still highly debated, and that our study cannot speak to the voracity of the δ^13^C data. However, this study may aid in the debate by providing a previously unrecognized, predictable mechanism by which extreme negative CIEs can be explained as a systematic global shift in δ^13^C_volc_ rather than secondary alteration. We also emphasize that the present model, despite including only an extremely simplified surface C cycle (see Materials and Methods), still captures the first-order long-term trends in atmospheric oxygen and δ^13^C_carb_. It does this by including the deep C cycle and specific differences in the cycling of carbonate and organic C following subduction. Thus, important messages in this contribution are that deep Earth processes play a vital role in mediating the surficial carbon cycle, and it is critical to consider the coupling between the surface and deep C cycles when investigating the long-term evolution of carbon and oxygen on Earth.

## Materials and Methods

### Modeling atmospheric oxygen levels and δ^13^C of carbonates

We used the carbon cycle model from Eguchi et al. (24), which tracks how a set of carbon reservoirs (C_i_) respond to changes in carbon fluxes (*F*_i_) among these reservoirs. The surface reservoirs are the atmosphere–ocean (C_atm_), inorganic carbonates (C_carb_), and organic carbon (C_org_; the model treats the ocean and atmosphere as a single reservoir). The transfer of carbon from the atmosphere–ocean reservoir into the carbonate and organic carbon reservoirs is controlled by a silicate weathering flux (*F*_w_, which comprises *F*_carb_ and *F*_org_). This flux is proportional to a constant for the strength of the silicate weathering feedback (*k*) and the amount of CO_2_ in the atmosphere–ocean (C_atm_). Carbon that is fluxed out of the atmosphere–ocean is deposited as either inorganic carbonate or organic carbon. To demonstrate that a CIE is possible without changing *f*_org_, we hold *f*_org_ constant at 0.20 throughout the model. To investigate whether the model can reproduce the proposed lower levels of atmospheric O_2_ during the Proterozoic, we add an oxidative weathering term (*F*_ow_) from Bergman et al. (55), which depends on a weathering constant (*k*_ow_) and a term for uplift (U).

The model has 3 different mantle reservoirs for C—primordial carbon (C_prm_), subducted carbonate (C_mcarb_), and subducted organic carbon (C_morg_). Primordial carbon, which existed in the mantle since Earth's accretion, receives no addition from subduction and is emitted at mid-ocean ridges (*F*_MORB_). Subducted carbonates and organic C have influxes from the surface through subduction (*F*_subc_ and *F*_subo_). Subducted carbon is released from mantle reservoirs via arc volcanoes (*F*_arc_) and ocean island volcanoes (*F*_OIB_). Carbon fluxes at arcs and ocean island volcanoes are a sum of C fluxes from the primitive mantle (*F*_arcp_ and *F*_OIBp_), subducted carbonates (*F*_arcc_ and *F*_OIBc_), and subducted organic C (*F*_arco_ and *F*_OIBo_). We prescribe variables to control the fraction of subducted carbonates (α_carb_) and organic carbon (α_org_) released at arc volcanoes, as well as variables to control the fraction of subducted carbonates (ε_carb_) and organic carbon (ε_org_) released at ocean islands. Eguchi et al. (24) found that C in carbonates is more efficiently released during subarc melting, while graphitized organic C is relatively refractory. Therefore, we treated carbonates as being completely released at arcs (α_carb_ = 1 and ε_carb_ = 0) and organic carbon as being completed released at ocean island volcanoes (α_org_ = 0 and ε_org_ = 1) when generating Figure 2 [see Figures S1–S7 (Supplementary Material) for model sensitivity to changes in these parameters].

A key feature of the model is the treatment of mantle residence times for the 2 forms of carbon. Subducted carbon released at arc volcanoes (predominantly inorganic carbonate) would have traveled on the order of hundreds of kilometers, while subducted carbon (predominantly graphitized organic C) released at ocean islands may have traveled to the deep mantle or core/mantle boundary on pathways of around 10,000 km before being re-emitted to the surface (24). If mantle convection occurs on the order of 1–10 cm/yr, subducted carbon will be released at arc volcanoes roughly 1–10 Myr after being subducted, while subducted carbon released at ocean island volcanoes will be released approximately 100–1,000 Myr after initial subduction. To account for these differences, we prescribe a timescale variable that controls how long subducted specific carbon pools remain in their respective mantle reservoir before being released at either arc volcanoes (τ_arc_) or ocean island volcanoes (τ_OIB_). By implementing these variables, the carbon emitted at arcs is proportional to the flux of carbon subducted τ_arc_ years ago, while carbon emitted at ocean island volcanoes is proportional to the flux of carbon subducted τ_OIB_ years ago. Additionally, the carbon emitted at arc volcanoes will have δ^13^C values influenced by inorganic (carbonate) C subducted τ_arc_ years ago, while the carbon emitted at ocean island volcanoes will have δ^13^C influenced organic C subducted τ_OIB_ years ago. This approach differs from traditional box models, in that it does not result in efficient mixing between carbon subducted at different times. Instead, it treats carbon cycling as something closer to a conveyor belt, where parcels of subducted lithologies retain the carbon inventory and δ^13^C signature they had at the time of their initial subduction rather than mixing with the entire mantle reservoir (Figure S9, Supplementary Material). This approach may be a better representation of what is occurring in nature because portions of the subducted slab that were recently subducted are spatially restricted from portions of the slab subducted much longer ago, and due to these spatial restrictions efficient mixing and equilibration seems unlikely (Figure S9, Supplementary Material). Finally, the total volcanic CO_2_ outgassing flux ${\rm{(}}{{\rm{F}}_{{\rm{out}}}})$ is a sum of the fluxes at arc volcanoes, ocean island volcanoes, and mid-ocean ridge volcanoes, and atmospheric oxygen levels are directly proportional to the mass of organic carbon accumulated in all surface and mantle reservoirs.

The system of equations representing the coupled surface and interior carbon cycle are:

(2) $$\begin{equation*}
\frac{{{{d}}{{\rm{C}}_{{\rm{atm}}}}}}{{{{dt}}}}={{{F}}_{{\rm{out}}}}\ ( t) + {F_{ow}}( t){\rm{ - }}{{{F}}_{\rm{w}}}( t).
\end{equation*}
$$

(3) $$\begin{equation*}
\frac{{{{d}}{{\rm{C}}_{{\rm{carb}}}}}}{{{{dt}}}}{\rm{ = \ (1 - }}{{{f}}_{{\rm{org}}}}{\rm{)}}{{{F}}_{\rm{w}}}( t){\rm{ - }}{{{F}}_{{\rm{subc}}}}( t).
\end{equation*}
$$

(4) $$\begin{equation*}
\frac{{d{C_{org}}}}{{dt}} = {f_{org}}{F_w}(t) - {F_{{\rm{subo}}}}(t) - {F_{ow}}(t).
\end{equation*}
$$

(5) $$\begin{equation*}
\frac{{{{d}}{{\rm{C}}_{{\rm{mcarb}}}}}}{{{{dt}}}}={{{F}}_{{\rm{subc}}}}\ (t){\rm{ - }}{{{F}}_{{\rm{arcc}}}}(t){\rm{ - }}{{{F}}_{{\rm{oibc}}}}(t).
\end{equation*}
$$

(6) $$\begin{equation*}
\frac{{{{d}}{{\rm{C}}_{{\rm{morg}}}}}}{{{{dt}}}}={{{F}}_{{\rm{subo}}}}\ (t){\rm{ - }}{{{F}}_{{\rm{arco}}}}(t)-{{{F}}_{{\rm{oibo}}}}(t).
\end{equation*}
$$

(7) $$\begin{equation*}
\frac{{{{d}}{{\rm{C}}_{{\rm{prm}}}}}}{{{{dt}}}}{\rm{ = - (}}{{{F}}_{{\rm{oibp}}}}{\rm{\ (}}t{\rm{) + }}{{{F}}_{{\rm{MORB}}}}(t){\rm{ + }}{{{F}}_{{\rm{arcp}}}}{\rm{(}}t{\rm{))}}.
\end{equation*}
$$

(8) $$\begin{equation*}
{{\rm{O}}_{{\rm{atm}}}}=\frac{{{C_{{\rm{org}}}} + {C_{{\rm{morg}}}}}}{{12.011}}\ 15.999*2.
\end{equation*}
$$

(9) $$\begin{equation*}
{{{F}}_{\rm{w}}}{\rm{(t)\ = \ k}}{{\rm{C}}_{{\rm{atm}}}}.
\end{equation*}
$$

(10) $$\begin{equation*}
{F_{carb}}( {\rm{t}}) = {F_w}( {\rm{t}}){\rm{ }}( {1 - {f_{org}}}).
\end{equation*}
$$

(11) $$\begin{equation*}
{F_{org}}( {\rm{t}}){\rm{ }} = {F_w}( {\rm{t}})( {{f_{org}}}).
\end{equation*}
$$

(12) $$\begin{equation*}
{{{F}}_{{\rm{subc}}}}( {\rm{t}}){\rm{\ = \ \chi }}{{{F}}_{{\rm{carb}}}}( {\rm{t}}).
\end{equation*}
$$

(13) $$\begin{equation*}
{{{F}}_{{\rm{subo}}}}( {\rm{t}}){\rm{\ = \ \chi }}{{{F}}_{{\rm{org}}}}( {\rm{t}}).
\end{equation*}
$$

(14) $$\begin{equation*}
{F_{{\rm{arc}}}}( {\rm{t}})\, = {F_{{\rm{arcc}}}}{\rm{\ (t) + }}{F_{{\rm{arco}}}}({\rm{t}}) + {F_{{\rm{arcp}}}}({\rm{t}}).
\end{equation*}
$$

(15) $$\begin{equation*}
{{{F}}_{{\rm{arcc}}}}( {\rm{t}})={{\rm{\alpha }}_{{\rm{carb}}}}\ {{{F}}_{{\rm{subc}}}}({\rm{t - }}{{\rm{\tau }}_{{\rm{arc}}}}{\rm{)}}.
\end{equation*}
$$

(16) $$\begin{equation*}
{{{F}}_{{\rm{arco}}}}( {\rm{t}})={{\rm{\alpha }}_{{\rm{org}}}}\ {{{F}}_{{\rm{subo}}}}({\rm{t - }}{{\rm{\tau }}_{{\rm{arc}}}}{\rm{)}}.
\end{equation*}
$$

(17) $$\begin{equation*}
{{{F}}_{{\rm{OIB}}}}( {\rm{t}})={{{F}}_{{\rm{OIBc}}}}{\rm{\ }}( {\rm{t}}){\rm{ + }}{{{F}}_{{\rm{OIBo}}}}( {\rm{t}}){\rm{ + }}{{{F}}_{{\rm{OIBp}}}}( {\rm{t}}).
\end{equation*}
$$

(18) $$\begin{equation*}
{{{F}}_{{\rm{OIBc}}}}( {\rm{t}})={\varepsilon _{{\rm{carb}}}}{{{F}}_{{\rm{subc}}}}\ ({\rm{t - }}{{\rm{\tau }}_{{\rm{OIB}}}}{\rm{)}}.
\end{equation*}
$$

(19) $$\begin{equation*}
{{{F}}_{{\rm{OIBo}}}}( {\rm{t}})={\varepsilon _{{\rm{org}}}}\ {{{F}}_{{\rm{subo}}}}{\rm{t - }}{{\rm{\tau }}_{{\rm{OIB}}}}{\rm{)}}.
\end{equation*}
$$

(20) $$\begin{equation*}
{{{F}}_{{\rm{out}}}}\ ( {\rm{t}}) = {{{F}}_{{\rm{OIB}}}}{\rm{\ }}( {\rm{t}}){\rm{ + }}{{{F}}_{{\rm{MORB}}}}( {\rm{t}}){\rm{ + }}{{{F}}_{{\rm{arc}}}}( {\rm{t}}).
\end{equation*}
$$

Figure S9 (Supplementary Material) is a schematic diagram of the box model described above. A main focus of the model is to demonstrate how δ^13^C_carb_ evolves in response to perturbations to carbon fluxes. Therefore, we track the δ^13^C evolution of different carbon reservoirs and fluxes with the following set of equations according to the assumptions outlined above:

(21) $$\begin{eqnarray*}
{\rm{\delta}}^{13} {\rm{C}}_{\rm{atm}}(t) &=& \frac{{{F}}_{\rm{OIB}}(t)} {{{F}}_{\rm{out}}(t)}\
{\rm{\delta }}^{\rm{13}} {\rm{C}}_{\rm{OIB}} (t) + \frac{{F}_{\rm{arc}} (t)} {{F}_{\rm{out}} (t)}
{\rm{\delta}}^{\rm{13}} {\rm{C}}_{\rm{arc}} (t) \nonumber \\
&& + \frac{{{{{F}}_{{\rm{MORB}}}}(t)}}{{{{{F}}_{{\rm{out}}}}(t)}}{{\rm{\delta }}^{{\rm{13}}}}{{\rm{C}}_{{\rm{MORB}}}}{{(t)}},
\end{eqnarray*}
$$

(22) $$\begin{equation*}
{{\rm{\delta }}^{{\rm{13}}}}{{\rm{C}}_{{\rm{OIB}}}}\ ( {{t}}) = \frac{{{{{F}}_{{\rm{OIBc}}}}(t)}}{{{{{F}}_{{\rm{OIB}}}}(t)}}\ {{\rm{\delta }}^{{\rm{13}}}}{{\rm{C}}_{{\rm{carb}}}}{\rm{(t - }}{{\rm{\tau }}_{{\rm{OIB}}}}{\rm{) + }}\frac{{{{{F}}_{{\rm{OIBo}}}}(t)}}{{{{{F}}_{{\rm{OIB}}}}(t)}}{{\rm{\delta }}^{{\rm{13}}}}{{\rm{C}}_{{\rm{org}}}}{\rm{(t - }}{{\rm{\tau }}_{{\rm{OIB}}}}{\rm{)\ + \ }}\nonumber\\
\times \,\frac{{{{{F}}_{{\rm{OIBp}}}}}}{{{{{F}}_{{\rm{OIB}}}}(t)}}{{\rm{\delta }}^{{\rm{13}}}}{{\rm{C}}_{{\rm{prm}}}},
\end{equation*}
$$

(23) $$\begin{equation*}
{{\rm{\delta }}^{{\rm{13}}}}{{\rm{C}}_{{\rm{arc}}}}\ ( {{t}}) = \frac{{{{{F}}_{{\rm{arcc}}}}(t)}}{{{{{F}}_{{\rm{arc}}}}(t)}}\ {{\rm{\delta }}^{{\rm{13}}}}{{\rm{C}}_{{\rm{carb}}}}{\rm{(t - }}{{\rm{\tau }}_{{\rm{arc}}}}{\rm{) + }}\frac{{{{{F}}_{{\rm{arco}}}}(t)}}{{{{{F}}_{{\rm{arc}}}}(t)}}{{\rm{\delta }}^{{\rm{13}}}}{{\rm{C}}_{{\rm{org}}}}{\rm{(t - }}{{\rm{\tau }}_{{\rm{arc}}}}{\rm{)}} \nonumber\\
+ \,\frac{{{{{F}}_{{\rm{arcp}}}}}}{{{{{F}}_{{\rm{arc}}}}(t)}}{{\rm{\delta }}^{{\rm{13}}}}{{\rm{C}}_{{\rm{prm}}}},
\end{equation*}
$$

(24) $$\begin{equation*}
{{\rm{\delta }}^{{\rm{13}}}}{{\rm{C}}_{{\rm{carb}}}}\ ( {{t}}) = {{\rm{\delta }}^{{\rm{13}}}}\ {{\rm{C}}_{{\rm{atm}}}}( {{t}}){\rm{ + 5}},
\end{equation*}
$$

(25) $$\begin{equation*}
{{\rm{\delta }}^{{\rm{13}}}}{{\rm{C}}_{{\rm{org}}}}\ ( {{t}}) = {{\rm{\delta }}^{{\rm{13}}}}\ {{\rm{C}}_{{\rm{atm}}}}( {{t}}){\rm{ - 20}},
\end{equation*}
$$

where ${{\rm{\delta }}^{{\rm{13}}}}{{\rm{C}}_{\rm{i}}}{\rm{(t)}}$ is the ${{\rm{\delta }}^{{\rm{13}}}}{{\rm{C}}_{\rm{i}}}$ value for carbon reservoir or flux i at time *t*, with atm = atmosphere–ocean reservoir, carb = carbonate reservoir, org = organic carbon reservoir, prm = primitive mantle reservoir, arc = arc flux, OIB = ocean island flux, and MORB = mid-ocean ridge flux. We set ${{\rm{\delta }}^{{\rm{13}}}}{{\rm{C}}_{{\rm{prm}}}}$ to a constant value of −5‰ for the duration of the model, as we have assumed it has no influx of carbon from the surface reservoirs, which leads to no changes to ${{\rm{\delta }}^{{\rm{13}}}}{{\rm{C}}_{{\rm{prm}}}}$ with time. The equations for ${{\rm{\delta }}^{{\rm{13}}}}{{\rm{C}}_{{\rm{crb}}}}( {{t}} ){\rm{\ }}$and ${{\rm{\delta }}^{{\rm{13}}}}{{\rm{C}}_{{\rm{org}}}}( {{t}} )$ are offset from ${{\rm{\delta }}^{{\rm{13}}}}{{\rm{C}}_{{\rm{atm}}}}( {{t}} )$ by +5 and −20, respectively, because we assume constant Δ^13^C_crb-org_ = −25‰ and *f*_org_ = 0.20.

The model run used to generate Figure 1 was designed to simulate increased strength of the silicate weathering feedback coupled with a shift to high fractions of C subduction (high χ). Figure S8 (Supplementary Material) illustrates how driving perturbations via changes in *k* differ from perturbations driving by changes in CO_2_ emissions. To simulate this scenario, the model was run with the initial conditions and parameters given in Table S1 (Supplementary Material). After evolving with no perturbations for 2.5 billion years, we prescribed that *k* instantaneously increased from 1 × 10^−9^ to 2 × 10^−3^ at 2.4 Ga (Figure 2e). We then prescribe that *k* linearly decays to 1 × 10^−5^ over a duration of 350 Ma. Coincident with the period on increased *k*, we increase χ from 0.1 to 0.99 to simulate a shift from continent-dominated sedimentation to ocean-dominated sedimentation and subduction. All subsequent increases in *k* are accompanied by the same increase in χ. To reproduce the relatively minor oxygenation and CIEs occurring in the Mesoproterozoic, we prescribe that *k* instantaneously increased from 1 × 10^−5^ to 2 × 10^−4^ at 1.4 Ga. We then prescribe that *k* linearly decays to 2 × 10^−5^ over a duration of 300 Ma. Finally, to reproduce the major oxygenation and CIEs of the Neoproterozoic, we prescribe that *k* instantaneously increased from 2 × 10^−5^ to 9 × 10^−4^ at 0.8 Ga. We then prescribe that *k* linearly decays to 7 × 10^−5^ over a duration of 200 Ma. At this point O_2_ levels are at ∼0.6 PAL. Therefore, we increase *k* to 1.2 × 10^−3^ linearly over a timescale of 100 Ma. The timescales of *k* decay are of same order of magnitude as Wilson cycle timescales, which we have assumed are controlling changes in the strength silicate weathering feedback. Sensitivity analysis shows that model results are relatively insensitive to changes in this parameter (see Figure S6, Supplementary Material). To better match the estimated duration of the different positive CIEs based on independent age models for the rock record, we also make minor changes to τ_OIB_ along with perturbations to *k*. Specifically, at 1.4 Ga, we change τ_OIB_ to 310 My, and at 0.8 Ga, we change τ_OIB_ to 230 My. We feel these minor changes to τ_OIB_ are justified because they lie well within the estimated range of mantle overturn times based on plate speeds of 1–10 cm/yr, as well as estimates of recycling times based on radiogenic isotopes (41). These minor variations in τ_OIB_ may reflect minor variations in rates of mantle convection and travel paths of subducted lithologies. These are the only prescribed model perturbations to generate Figure 1 of the main text.

## Data Sharing Plan

Code for model will be uploaded to corresponding author's github.

## Supplementary Material

pgac036_Supplemental_File

## Data Availability

Model code used to generate Figure 2 are available on Github (https://github.com/jameseguchi/Eguchi_et_al_2022).
