# Supplementary material for: Proterozoic supercontinent break-up as a driver for oxygenation events and subsequent carbon isotope excursions
Source: PNAS Nexus. 2022 Mar 30;1(2):pgac036. doi: 10.1093/pnasnexus/pgac036 (PMC9802223; doi:10.1093/pnasnexus/pgac036)
Supplement: pgac036_Supplemental_File [file pgac036_supplemental_file.docx]

**Supplementary Information**

**­­­Proterozoic supercontinent break-up as a driver of oxygenation events and subsequent carbon isotope excursions**

James Eguchi^1^, Charles W. Diamond^1^, Timothy W. Lyons^1^

^1^University of California Riverside, Department of Earth and Planetary Sciences, Riverside, CA, USA

**Tables**

**Table S1** Model initial condition and parameter values

| **Initial Conditions** | **Value** | **Units** |
| --- | --- | --- |
| C_atm_(0) | 0 | [g] |
| C_carb_(0) | 0 | [g] |
| C_org_(0) | 0 | [g] |
| C_mcarb_(0) | 0 | [g] |
| C_morg_(0) | 0 | [g] |
| C_prm_(0) | 10^23^ | [g] |
| *F*_MORB_, *F_arcp_*, *F_oibp_* | 10^18^ | [g/Myr] |
| *δ^13^C_atm_* | -5 | [‰] |
| *δ^13^C_carb_* | 0 | [‰] |
| *δ^13^C_org_* | -25 | [‰] |
| **Parameters** |  |  |
| *f*_org_ | 0.2 | - |
| *k* | 1×10^-9^ | [Myr^-1^] |
| *k*_ow_ | 9.3×10^19^ | [g/Myr] |
| *U* | 1 | - |
| *χ* | 0.1 | - |
| α_carb_ | 1 | - |
| α_org_ | 0 | - |
| *ε*_carb_ | 0 | - |
| *ε*_org_ | 1 | - |
| δ^13^C_prm_ | -5 | - |
| *τ*_arc_ | 30 | Myr |
| *τ_OIB_* | 310 | Myr |
| *dt* | 1 | Myr |

**Sensitivity Analysis**

Here we test the sensitivity of the model to changes in the various parameters. Since a major contribution of this work is to provide an alternative interpretation of extreme CIEs, we emphasize parameters that affect the evolution of δ^13^C_Carb_ in our model. We focus on the time interval of 1000 to 400 Ma to compare how our model results agree with the broad positive CIE seen in the record during this interval and the following extremely negative CIE.

In Figure S1, we explore the effects of changing the magnitude of the increase in *k* (strength of silicate weathering feedback). As the magnitude of the increase in *k* becomes larger, the sizes of both the positive and negative CIE also increase. As the magnitude of the increase in *k* becomes larger, the subduction flux also becomes larger, resulting in larger arc and ocean island fluxes. An interesting result arises from the large *k* increase scenario: the δ^13^C curve shows greater oscillations as *k* increases. Large oscillations have been observed in the Neoproterozoic δ^13^C_carb_ record (1). The oscillations in the model are a result of rapid decay in the weathering flux after initial *k* increase, which is followed by an increase in the weathering flux when subducted C is recycled at arcs due to increased CO_2_ outgassing. The model suggests that there should be oscillations in δ^13^C_carb_ due to C recycling at arcs—an observation that may deserve more attention in the future.

Another interesting feature is that as the magnitude of *k* increases, the duration of the extremely negative CIE predicted for the Ediacaran decreases. As *k* increases, the silicate weathering feedback becomes more efficient, and the decrease in the weathering flux occurs over a shorter time interval. As a result, the spike of organic C release at ocean islands occurs over a shorter time period, making the predicted negative excursion shorter. This relationship may help reconcile the model results with the growing body of increasingly precise time constraints for the Ediacaran Shuram anomaly (2).


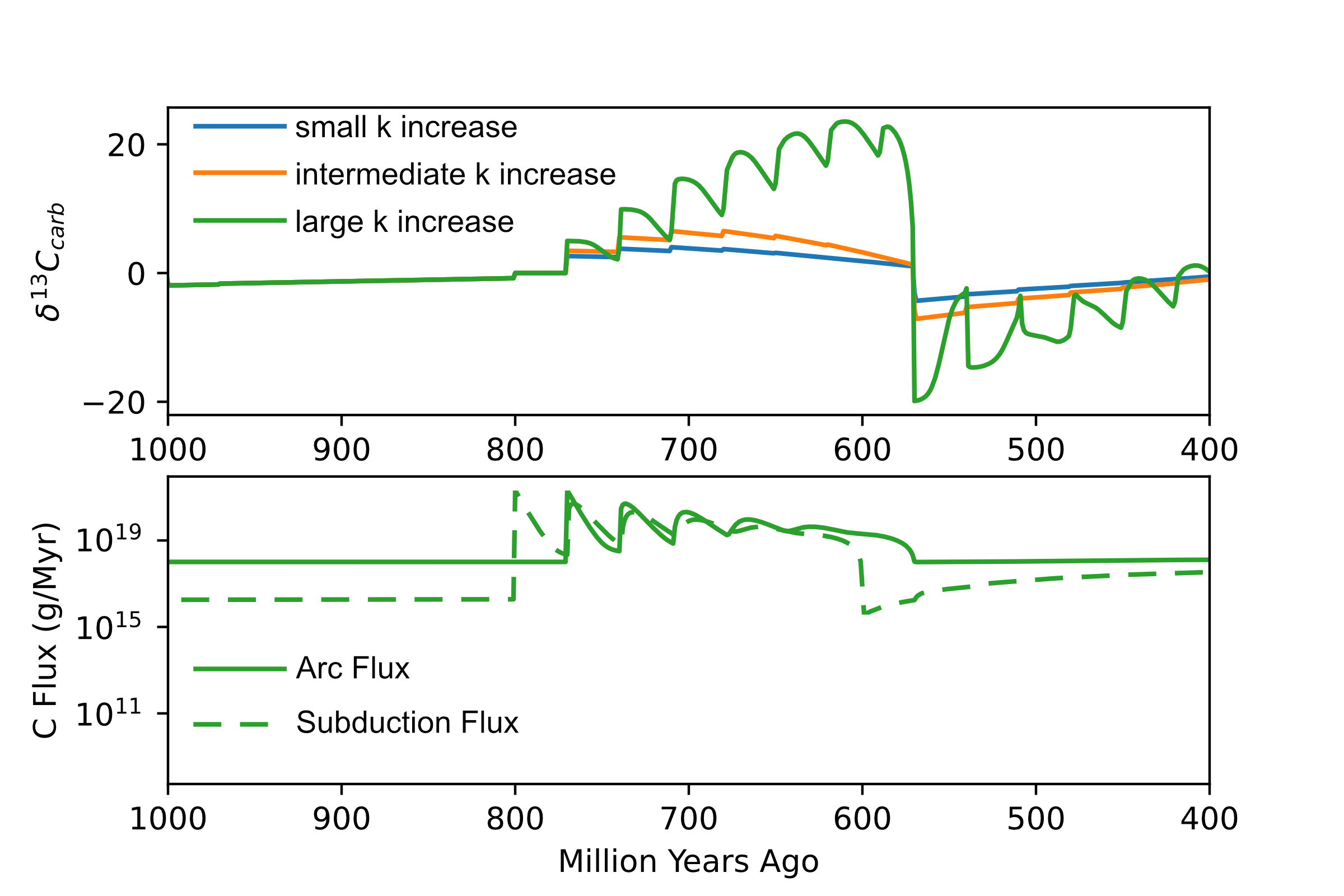


**Figure S1** Sensitivity of model to changes in the magnitude of *k* increase.

Figure S2 shows how changing the magnitude of the *k* decay affects the model results. We see that as *k* decays to lesser extents, the magnitude of the positive CIE increases, while the magnitude of the negative CIE decreases. As the magnitude of the *k* decay decreases, the weathering flux remains higher, resulting in the subduction flux remaining at higher values. A larger C subduction flux translates to higher carbonate release at arcs, which results in a positive CIE with a larger magnitude. Since the arc flux remains higher with a smaller *k* decay, the difference between the arc flux and the ocean island flux when the spike of subducted organic C is initially released at ocean islands is smaller, resulting in a smaller magnitude negative CIE.


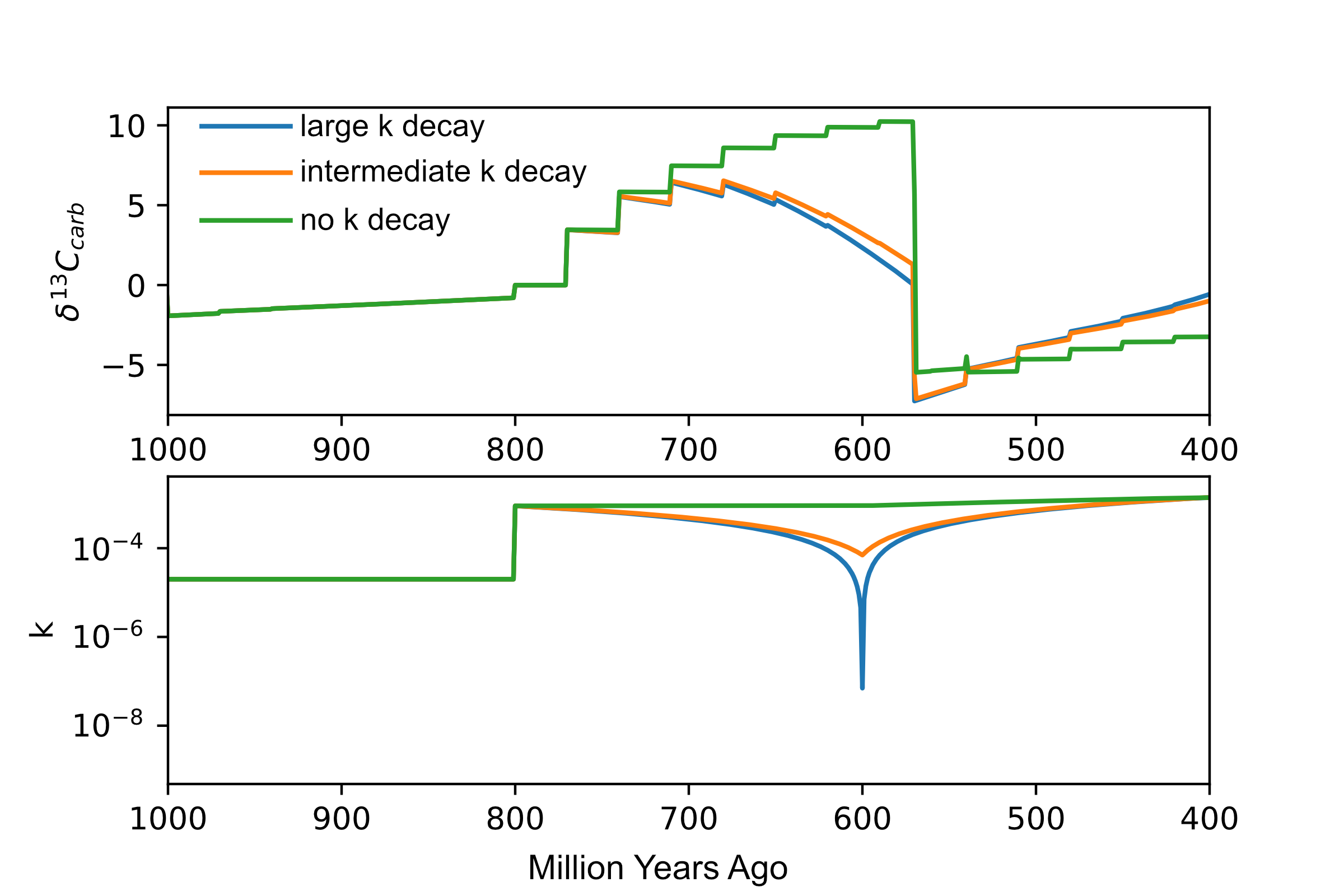


**Figure S2** Sensitivity of model to changes in the magnitude of *k* decay.

Figure S3 shows how the model responds to changes in the fraction of buried carbon that is subducted into the mantle (χ). As a higher fraction of buried carbon is subducted into the mantle rather than remaining in the crustal reservoir (larger χ), we see that the magnitude of the positive and negative CIEs increase. This outcome is simply because as more C is subducted, the release of carbonate-derived CO_2­_ outgassed at arcs increases, which results in higher δ^13^C_volc_ and thus increases the magnitude of the modeled positive CIE. Subsequently, the CO_2_ at ocean islands will also be larger resulting in a larger negative CIE.


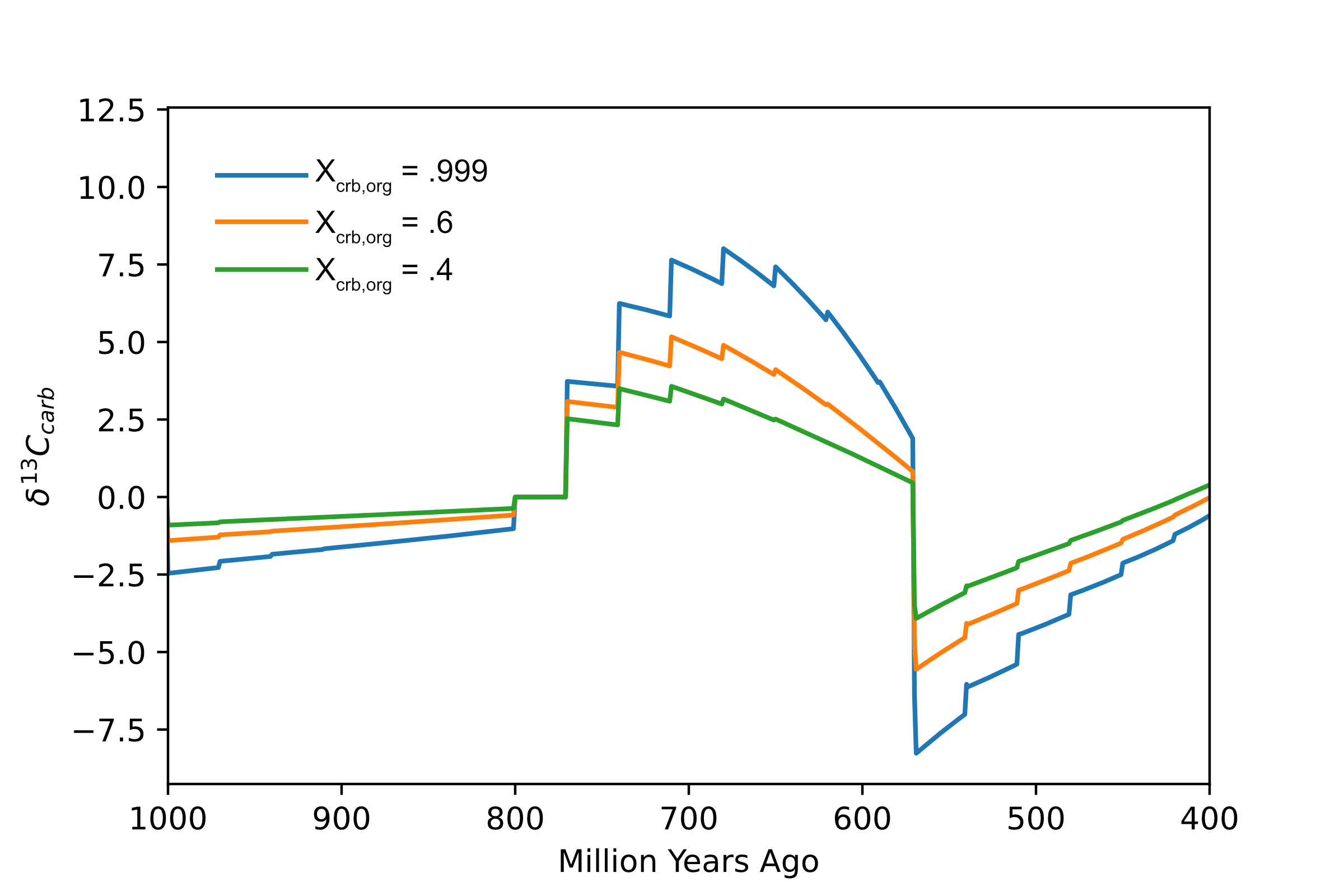


**Figure S3** Sensitivity of model to changes in fraction of buried carbon that is subducted into the mantle.

Figure S4 shows the sensitivity of the model to changes in the fraction of subducted carbonate outgassed at arcs (α_carb_). As the fraction of subducted carbonates outgassed at arcs increases, the magnitudes of the positive and negative CIEs increase. The magnitude of the positive CIE increases because as more subducted carbonates are outgassed at arcs, the higher the δ^13^C of CO_2_ outgassed and the greater the magnitude of the positive CIE. The negative CIE increases in magnitude with higher α_carb_ because as α_carb_ increases, less carbonate is subducted deep into the mantle and, therefore, less subducted carbonate is outgassed at ocean islands. The result is organic C outgassing at ocean islands dominates, resulting in a greater magnitude negative CIE.


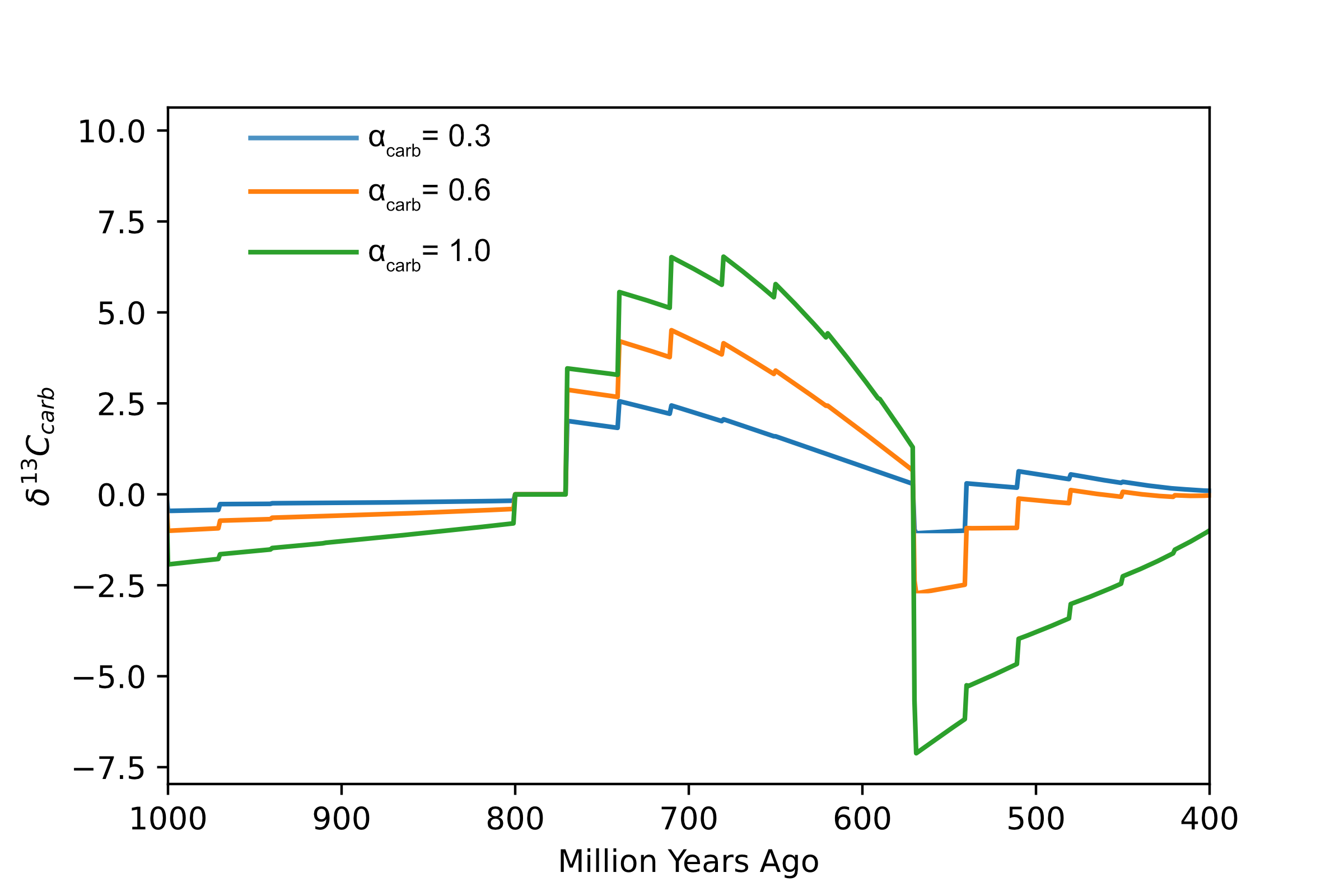


**Figure S4** Sensitivity of model to changes in the fraction of subducted carbonates released at arc volcanoes.

Figure S5 shows the sensitivity of the model to changes in the fraction of subducted organic C outgassed at arc volcanoes (α_org_). As α_org_ increases, the magnitude of the positive and negative CIEs decrease. The magnitude of the positive CIE decreases when the fraction of subducted organic C outgassed at arcs increases. Outgassing more organic C at arc volcanoes decreases δ^13^C of CO_2_ outgassed at arcs, thus decreasing the magnitude of the positive CIE. Increasing α_org_ decreases the magnitude of the negative CIE because increased α_org_ releases more subducted organic C at arcs, which decreases the amount of organic C available to be outgassed at ocean islands. The decrease in the amount of organic C outgassed at arcs results in higher δ^13^C of CO_2_ outgassed at ocean islands, resulting in a smaller magnitude negative CIE.


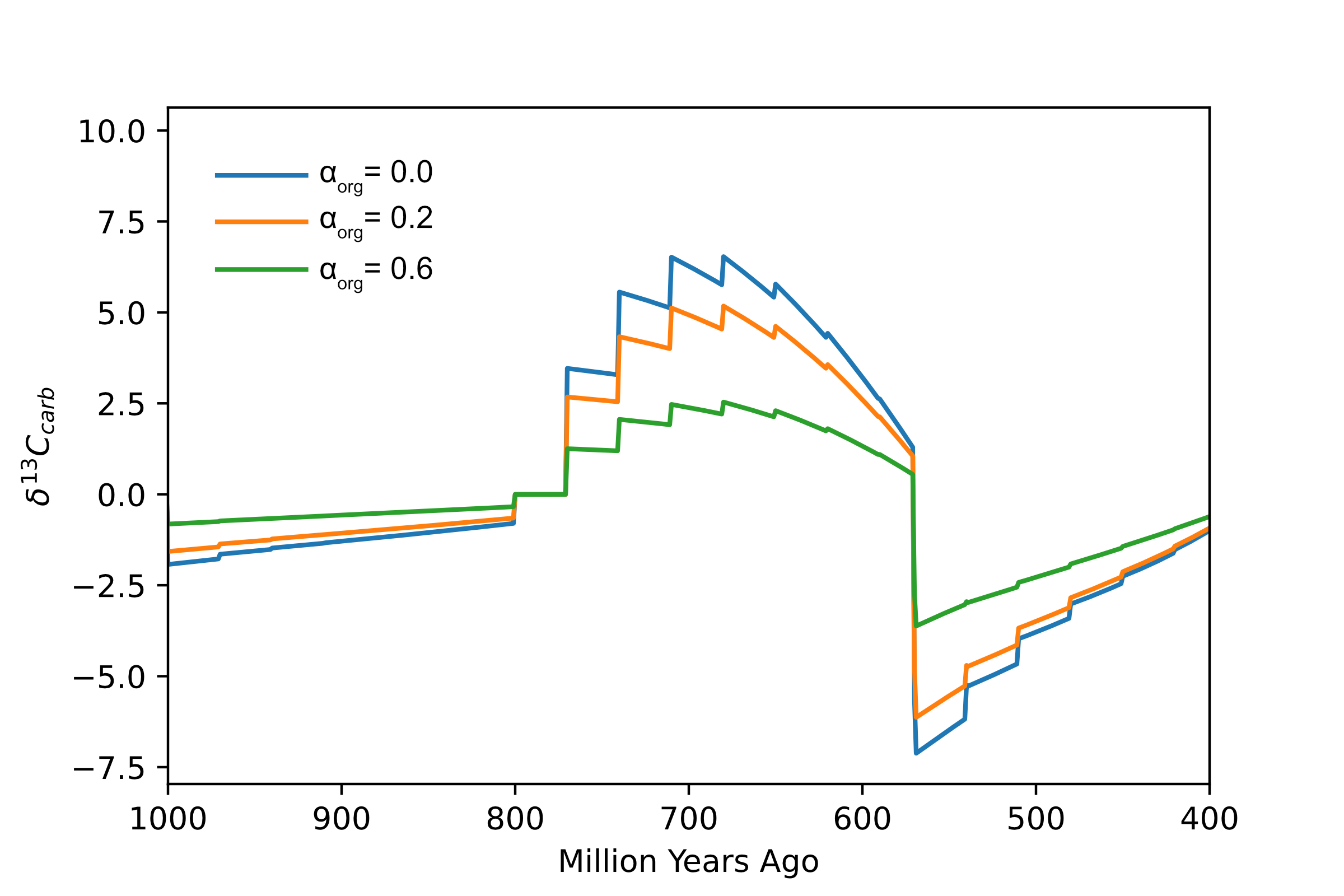


**Figure S5** Sensitivity of model to changes in the fraction of subducted organic C released at arc volcanoes.

Figure S6 shows the sensitivity of the model to changes in the decay time of *k*. Results show that as the *k* decay time increases, the duration of the positive and negative CIEs also increase. Increased *k* decay times increase the duration of CIEs simply because the weathering flux remains higher for a longer time, resulting in a longer duration of enhanced release of subducted C at both arcs and ocean islands.


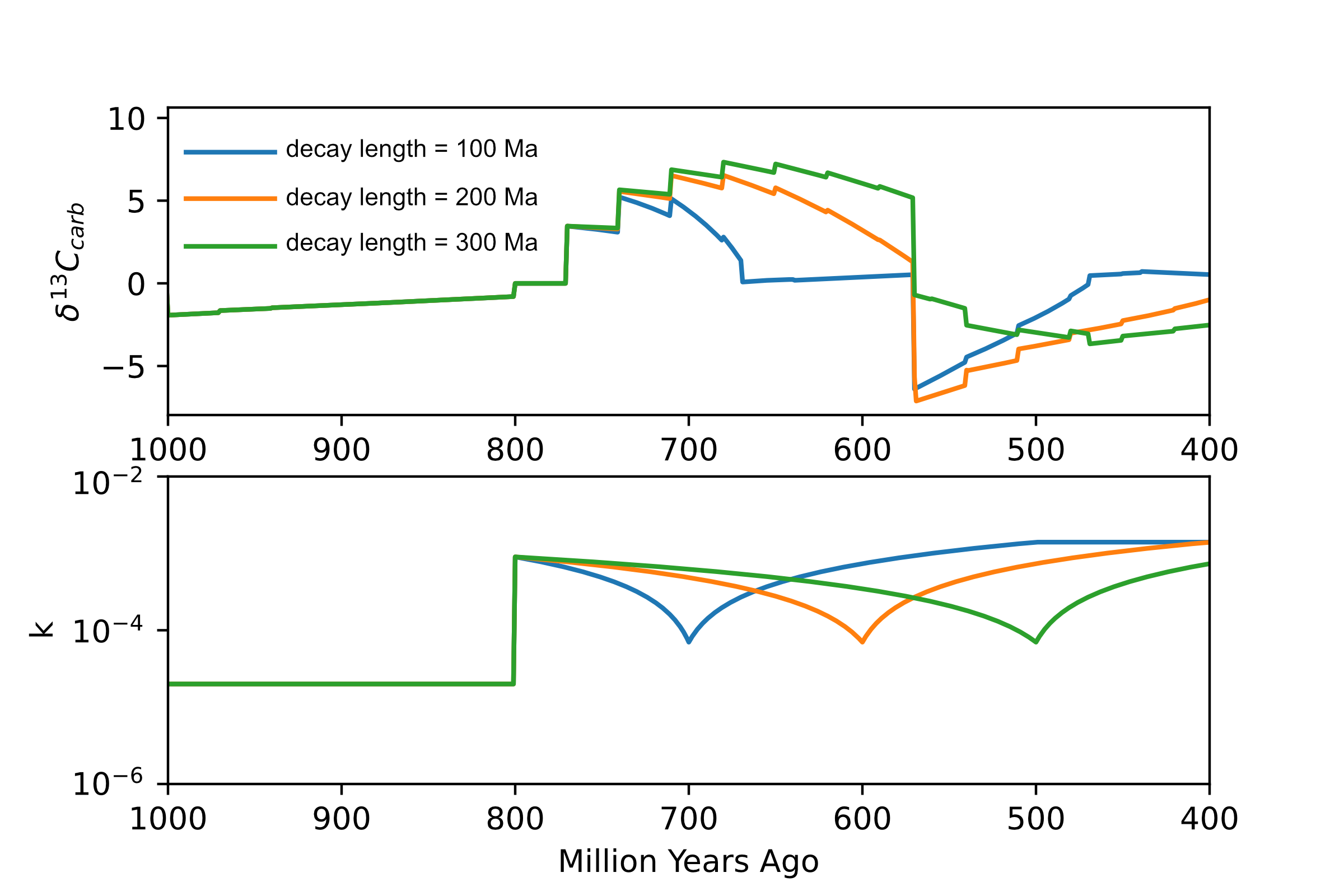


**Figure S6** Sensitivity of model to changes in length of *k* decay time.

Figure S7 shows the sensitivity of the model to changes in the residence time of organic C in the mantle (τ_org_). As τ_org_ increases, the length of the positive CIE increases because the positive CIE only ends when organic C is released at ocean islands. Therefore, when organic C resides in the mantle longer, the positive CIE is longer-lived.


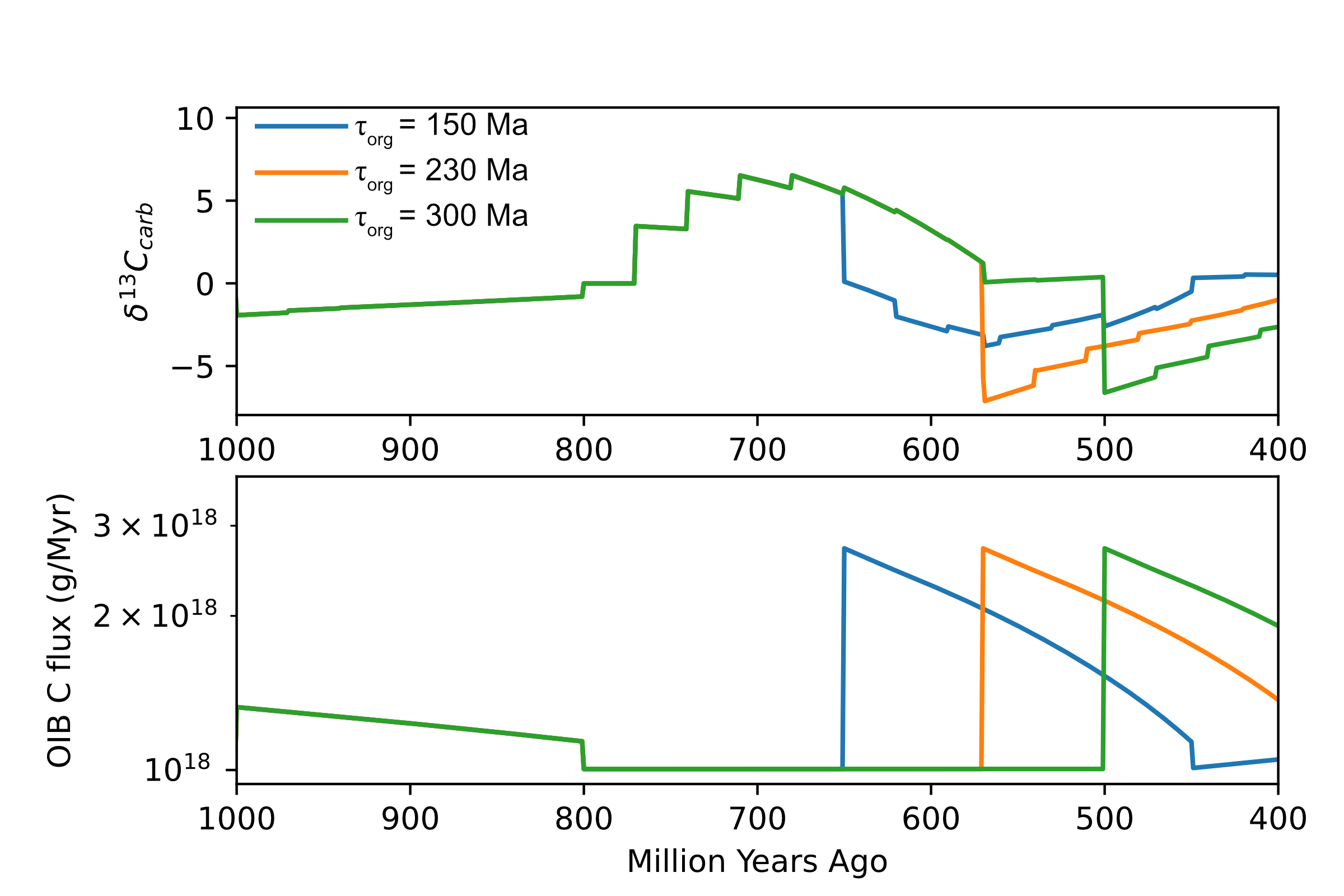


**Figure S7** Sensitivity of model to changes in the mantle residence time of organic C.

**CO_2_ emissions versus weathering**

The perturbation to the carbon cycle in Eguchi et al. (3), which was argued to have spawned the GOE oxygenation and Lomagundi-Jatuli CIE, was enhanced degassing of CO_2_ and concomitant enhanced continental weathering, and ultimately C deposition in the marine system as expressed in the simple equation:

F_w_ = *k* × [CO_2,atm_], (1)

where F_w_ is the weathering flux of silicate rocks, *k* is a scalar that captures the strength of the weathering feedback (4), and [CO_2,atm_] is the concentration of CO_2_ in the atmosphere. Equation (1) shows that the weathering flux is not only sensitive to changes in CO_2_ emissions but also to changes to the strength of the weathering feedback (*k*).

In the main text, we investigate the effects of changing *k* on the evolution of atmospheric oxygen, CO_2_, and the carbon isotope values recorded in marine carbonates. The parameter *k* can be thought of as the variable that tracks the sensitivity of silicate weathering to changes in CO_2_ and is modulated by various factors such as lithology, continental relief, continental area, runoff, paleogeography, and plant coverage at younger times (4). We use the model presented in Eguchi et al. (3) to examine the relative differences via changing *k* versus changing CO_2_ fluxes (see methods for more details). In the left column of Figure S8, perturbations are driven by a prescribed increase in CO_2_ flux from mid-ocean ridges (Fig. S8e), while the perturbation in the right column is driven by a prescribed increase in *k* (Fig. S8j). Figure S8 illustrates several key differences between driving the carbon cycle perturbation via enhanced CO_2_ emissions versus a strengthened weathering feedback. 1) When CO_2_ emissions are increased, the result is an increase in the atmospheric CO_2_ reservoir (Fig. S8g), while an increase in *k* results in a decrease in atmospheric CO_2_ (Fig. S8h). If enhanced carbon burial is driven by a stronger weathering feedback, then CO_­2_ is drawn out of the atmosphere at a faster rate without a commensurate increase in CO_2_ emissions, leading to a decrease in the atmospheric CO_2_ reservoir. It is widely believed that CO_2_ concentration in the atmosphere has decreased through time (5–7). The decrease in atmospheric CO_2_ through time has been attributed to the impact of a warming sun on the silicate weathering feedback. However, if the strength of the silicate weathering feedback has steadily increased through time, perhaps due to steady increases in subaerial continental area, then CO_2_ decrease with time may, in part, be due to increases in *k* over time. 2) The differing evolution of the atmospheric CO_2_ reservoirs also affects how carbon burial fluxes evolve. When CO_2_ emissions are increased, the result is an increased atmospheric CO_2_ reservoir, which causes a permanent increase in the weathering flux for as long as elevated CO_2_ emissions are sustained (Fig S8e). In contrast, when the perturbation is driven by an enhanced *k*, the carbon burial flux will initially increase due to increased *k* in eqn. 1. A decrease in *k* decreases the size of the atmospheric CO_2_ reservoir as discussed above. Due to the decreased size of the atmospheric CO_2_ reservoir, the weathering flux will also decrease because that flux is dependent on the concentration of CO_2_ in the atmosphere (eqn. 1) (Fig S8f). 3) The different behaviors of weathering fluxes between the two scenarios yield important differences in the evolution of δ^13^C of marine carbonates. In the case of the CO_2_ emission-driven scenario, there is a positive CIE that terminates with a steady-state of near-zero δ^13^C values (Fig. S8a). In the *k*-driven scenario, there is a positive CIE followed by a negative CIE. This difference in behavior results from differences in CO_2_ fluxes at different volcanic sites (arc vs. ocean islands). In the CO_2_-driven scenario, the arc CO_2_ flux remains elevated after the initial increase. The subducted C flux remains high due to a sustained increase in the weathering flux (Fig. S8e). Therefore, the delayed increase in CO_2_ emissions at ocean islands balances out the increased flux at arc volcanoes (Fig. S8e), bringing the overall balance of δ^13^C of CO_2_ emissions back to around -5 ‰ and resulting in near-zero δ^13^C for marine carbonates (Fig. S8a).

In contrast, the arc CO_2_ flux in the *k*-driven scenario decreases after its initial increase because the flux of subducted carbon decreases with the decrease in weathering flux described above. The trend of an initial spike of CO_2_ released at arcs followed by a decrease is mirrored in the ocean island flux. However, since the CO_2_ flux at arc volcanoes has already decayed, the ocean island CO_2_ flux exceeds the CO_2_ flux at arcs for a short time (Fig. S8f). This relationship shifts the δ^13^C of CO_2_ emissions to values less than - 5 ‰, resulting in a negative CIE recorded in marine carbonates (Fig. S8b). This interesting behavior has been observed in the isotopic record of the Paleoproterozoic as the Lomagundi-Jatuli (8) followed by the Shunga-Francevillian (9–12) and during the Neoproterozoic as a prolonged positive CIE followed by the Shuram anomaly (13).

**
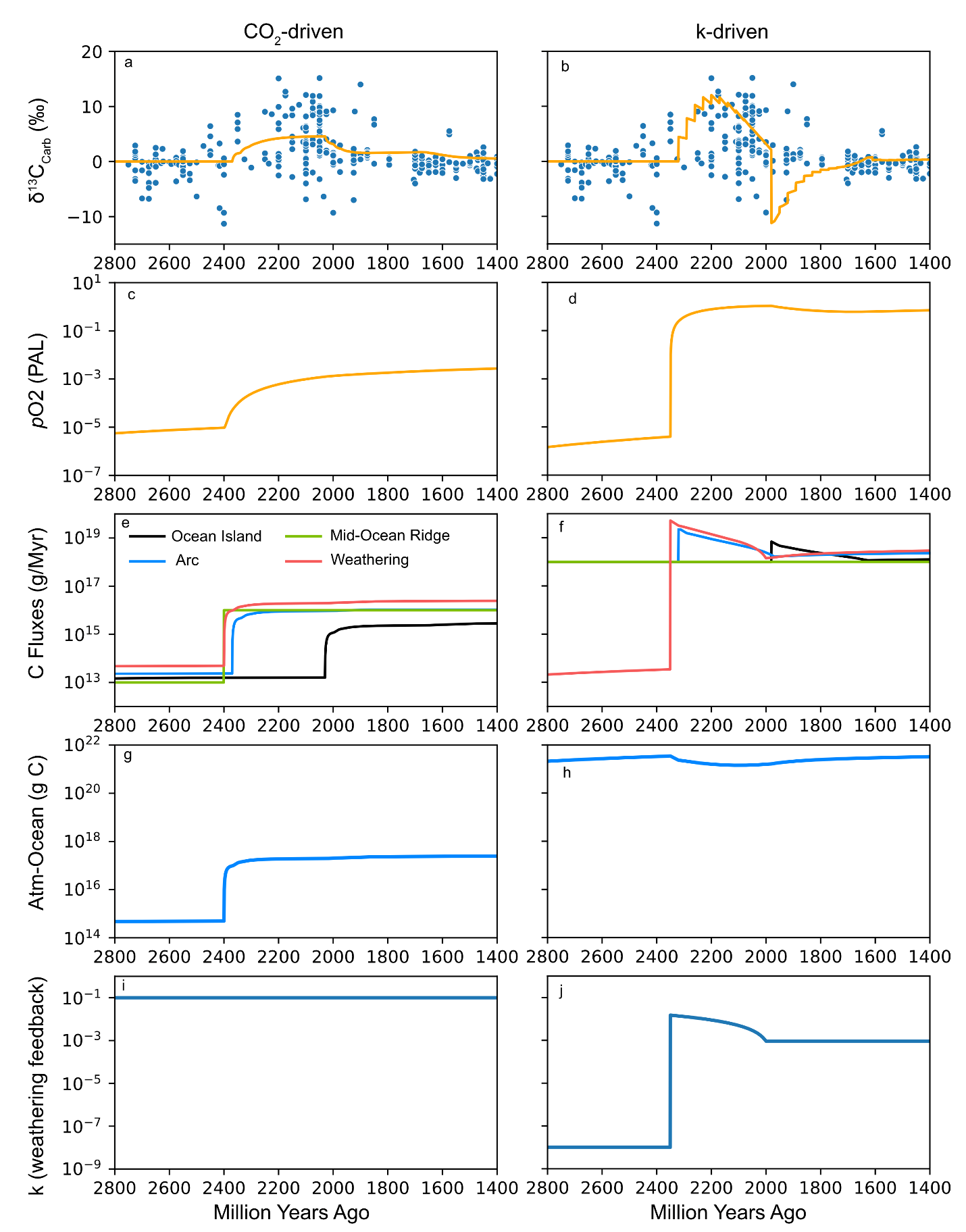
**

**Figure S8** Comparison of CO_2_-driven versus weathering-driven model perturbations. **a**, δ^13^C of marine carbonates versus time, with blue symbols representing measurement (14) and orange curve representing model results. CIE is generated by prescribing increased MORB CO_2_ emissions. **b,** Same as **a** but CIE is generated by prescribing an increase in *k*. **c,d,** Model evolution of atmospheric O_2_ levels (relative to present (PAL)) versus time. **e,** evolution through time of C fluxes of different volcanic settings and C drawdown flux from atmosphere driven by silicate weathering. An increase in MORB CO_2_ emissions was prescribed in the model, all other fluxes evolved according to differential equations outlined in text. **f**, same as **e** except MORB CO_2_ emissions were held constant throughout the model. **g,h,** Model evolution of the ocean-atmosphere reservoir of C through time. **i,j,** prescribed *k* (weathering feedback strength) in model versus time. *k* is held in constant model runs for **a, c, e, g,** and **i**. There is a prescribed in increase in *k* followed by a linear decrease in *k* to an intermediate value in **b, d, f, h,** and **j.**


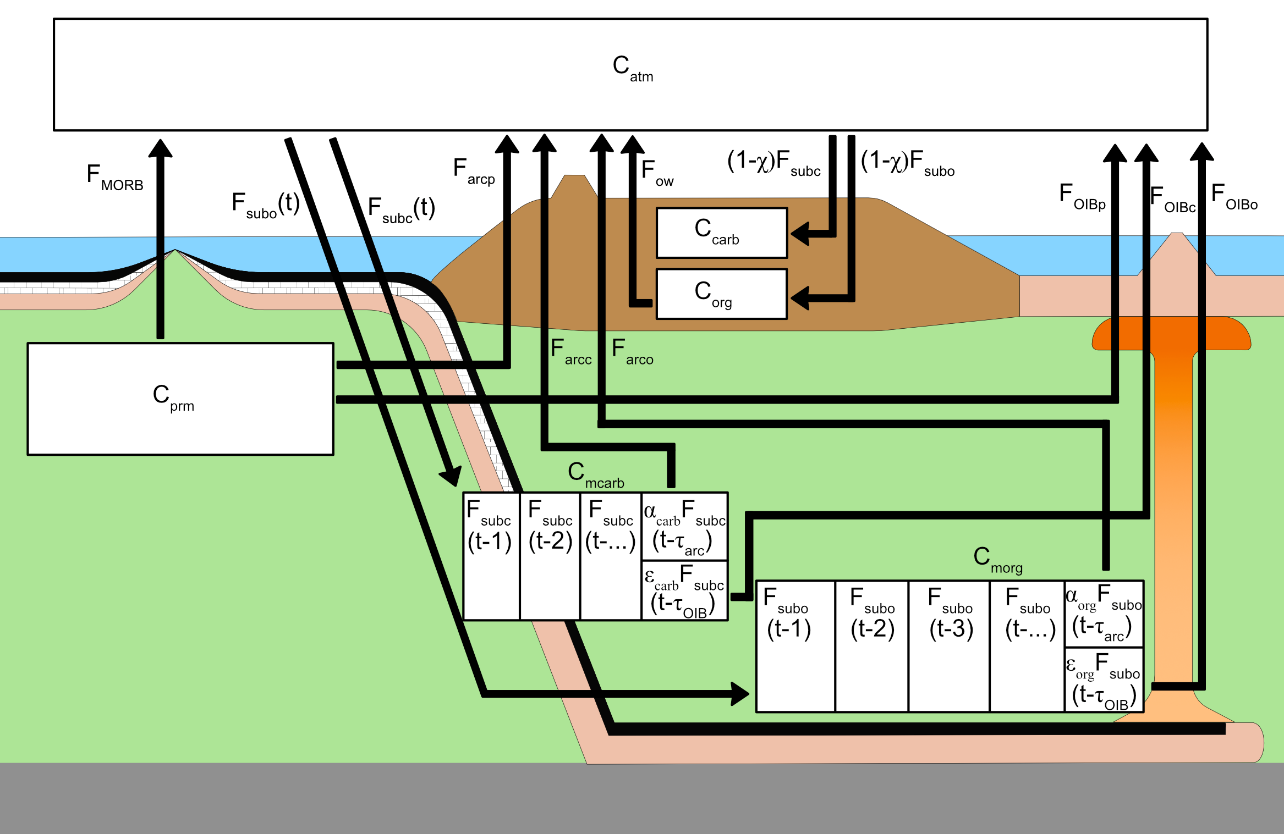


**Figure S9** Schematic diagram of box model. Boxes represent carbon reservoirs while arrows represent carbon fluxes. The mantle carbonate (C_mcarb­_) and mantle organic C (C_morg_) reservoirs are partitioned into parcels subducted at differing time steps within in the model. This is to illustrate that in this model parcels of rock subducted at different times do not efficiently mix, but rather retain the fluxes and δ^13^C values that they had upon initial subduction. Ultimately, when subducted carbon is released at either arc volcanoes or ocean island volcanoes, the fluxes and δ^13^C have values at τ_arc_ or τ_OIB_ years ago. See methods for model description.

**References Cited**

1. G. P. Halverson, P. F. Hoffman, D. P. Schrag, A. C. Maloof, A. H. N. Rice, Toward a Neoproterozoic composite carbon-isotope record. *Bull. Geol. Soc. Am.* **117**, 1181–1207 (2005).

2. A. D. Rooney, M. D. Cantine, K. D. Bergmann, I. Gómez-pérez, B. Al, Calibrating the coevolution of Ediacaran life and environment. 1–7 (2020).

3. J. Eguchi, J. Seales, R. Dasgupta, Great Oxidation and Lomagundi events linked by deep cycling and enhanced degassing of carbon. *Nat. Geosci.* **13**, 71–76 (2020).

4. J. K. Caves, A. B. Jost, K. V. Lau, K. Maher, Cenozoic carbon cycle imbalances and a variable weathering feedback. *Earth Planet. Sci. Lett.* **450**, 152–163 (2016).

5. D. C. Catling, K. J. Zahnle, The Archean atmosphere. *Sci. Adv.* (2020).

6. C. Sagan, G. Mullen, Earth and Mars: Evolution of atmospheres and surface temperatures. *Science (80-. ).* **177**, 52–56 (1972).

7. J. F. Kasting, Faint young Sun redux. *Nature* **464**, 687–689 (2010).

8. A. Bekker, J. A. Karhu, A. J. Kaufman, Carbon isotope record for the onset of the Lomagundi carbon isotope excursion in the Great Lakes area, North America. *Precambrian Res.* **148**, 145–180 (2006).

9. L. R. Kump, *et al.*, Isotopic evidence for massive oxidation of organic matter following the great oxidation event. *Science (80-. ).* **334**, 1694–1696 (2011).

10. A. E. Črne, *et al.*, Petrography and geochemistry of carbonate rocks of the Paleoproterozoic Zaonega Formation, Russia: Documentation of 13C-depleted non-primary calcite. *Precambrian Res.* **240**, 79–93 (2014).

11. V. A. Melezhik, A. E. Fallick, A. T. Brasier, A. Lepland, Carbonate deposition in the Palaeoproterozoic Onega basin from Fennoscandia: A spotlight on the transition from the Lomagundi-Jatuli to Shunga events. *Earth-Science Rev.* **147**, 65–98 (2015).

12. K. Motomura, *et al.*, Redox fluctuation and δ13Corg-δ34S perturbations recorded in the 1.9 Ga Nuvilik Formation of the Cape Smith belt, Canada. *Precambrian Res.* **359**, 106191 (2021).

13. J. P. Grotzinger, D. A. Fike, W. W. Fischer, Enigmatic origin of the largest-known carbon isotope excursion in Earth’s history. *Nat. Geosci.* **4**, 285–292 (2011).

14. J. Krissansen-Totton, R. Buick, D. C. Catling, A statistical analysis of the carbon isotope record from the Archean to phanerozoic and implications for the rise of oxygen. *Am. J. Sci.* **315**, 275–316 (2015).
